# Supplementary material for: Curcumin-Rich Diet Mitigates Non-Alcoholic Fatty Liver Disease (NAFLD) by Attenuating Fat Accumulation and Improving Insulin Sensitivity in Aged Female Mice under Nutritional Stress
Source: Biology (Basel). 2024 Jun 26;13(7):472. doi: 10.3390/biology13070472 (PMC11274271; doi:10.3390/biology13070472)

Supplementary file

Curcumin-Rich Diet Mitigates Non-Alcoholic Fatty Liver Disease (NAFLD) by Attenuating Fat accumulation and Improving Insulin Sensitivity in Aged Female Mice Under Nutritional Stress

Gopal Lamichhane [1], Da-Yeon Lee [1], Rienna Franks[1], Femi Olawale [1], Jong-Beom Jin [1], Josephine M. Egan [2], and Yoo Kim [1,*]

[1] Department of Nutritional Sciences, Oklahoma State University, Stillwater, OK 74078, USA
[2] Laboratory of Clinical Investigation, National Institute on Aging, Baltimore, MD 21224, USA

* Correspondence: yoo.kim@okstae.edu; Tel.: (optional; include country code; if there are multiple corresponding authors, add author initials)

Table S1. Sequence of primer used for real-time RT-PCR.

S.N.	Primer	Sequence (5′-3′)
1.	ACC1	F-ATTGGGACCCCAGAGCTA
		R-CCCGCTCCTTCAACTTGCT
2.	ACC2	F-GGGCTCCCTGGATGACAAC
		R-TTCCGGGAGGAGTTCTGGA
3.	PPARα	F-GCAGCTCGTACAGGTCATCA
		R-CTCTTCATCCCCAAGCGTAG
4.	PPARγ	F-ATGCCAAAAATATCCCTGGTTTC
		GGAGGCCAGCATGGTGTAGA
5.	ChREBP	F-CTGGGGACCTAAACAGGAGC
		R-GAAGCCACCCTATAGCTCCC
6.	SREBP	F-GCGGTTGGCACAGAGCTT
		R-GGACTTGCTCCTGCCATCAG
7.	LPL	FTTTTCTGGGACTGAGGATGG
		GTCAGGCCAGCTGAAGTAGG
8.	β-actin	F-TACCACCATGTACCCAGGCA
		R-CTCAGGAGGAGCAATGATCCTGAT
9.	18S	F-CGATCCGAGGGCCTCATCA
		R-AGTCCCTGCCCTTTGTACACA

Figure S1. Detail of Western blot membrane.

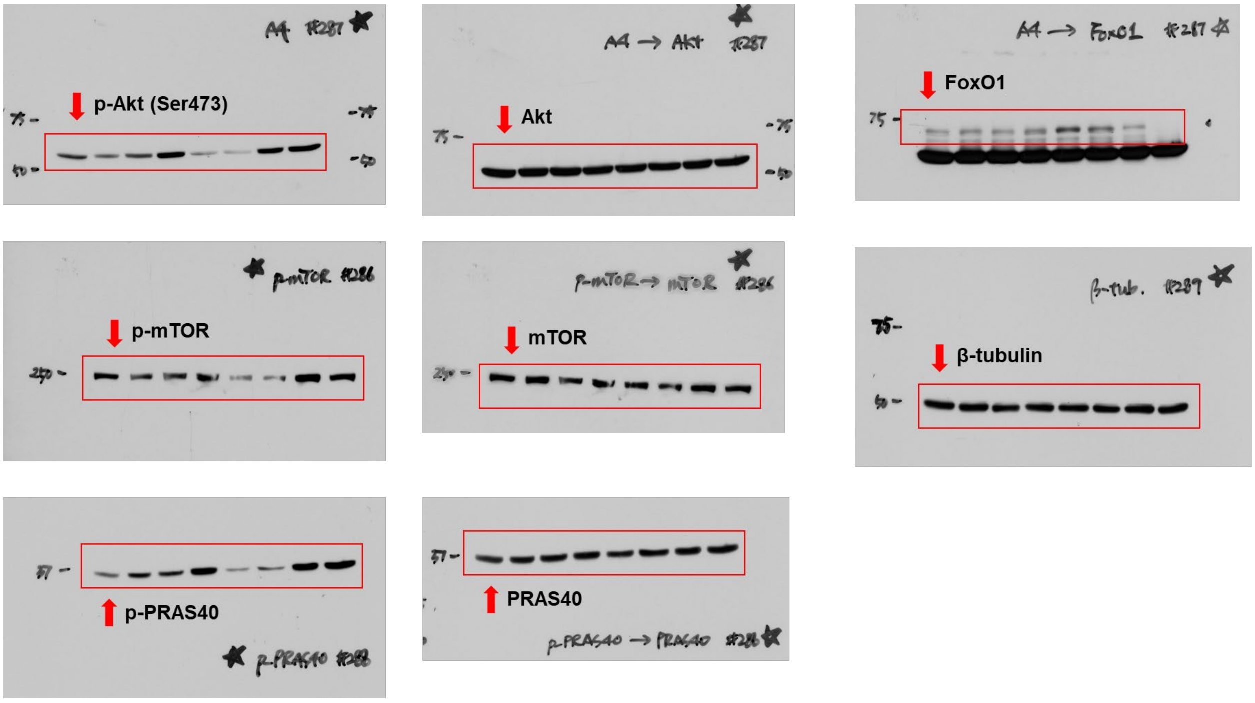

Supplement: Supplementary file 1 [file biology-13-00472-s001.zip › biology-3041304-supplementary.pdf]
