# Supplementary material for: Curcumin-Rich Diet Mitigates Non-Alcoholic Fatty Liver Disease (NAFLD) by Attenuating Fat Accumulation and Improving Insulin Sensitivity in Aged Female Mice under Nutritional Stress
Source: Biology (Basel). 2024 Jun 26;13(7):472. doi: 10.3390/biology13070472 (PMC11274271; doi:10.3390/biology13070472)
Supplement: Supplementary file 1 [file biology-13-00472-s001.zip › biology-3041304-supplementary.pptx]

## Slide 1
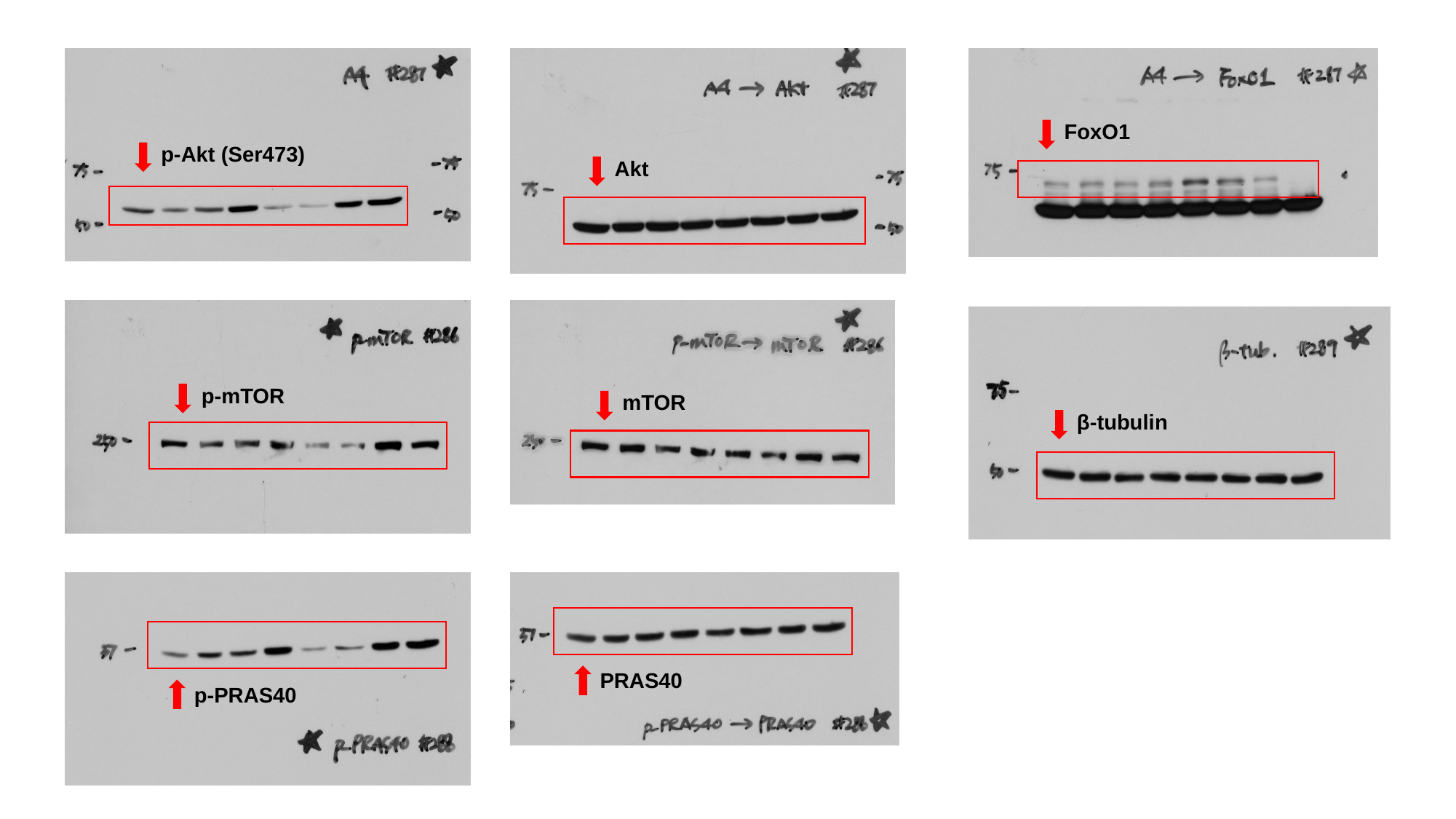

FoxO1
p-Akt (Ser473)
Akt
p-mTOR
mTOR
β-tubulin
PRAS40
p-PRAS40

## Slide 2
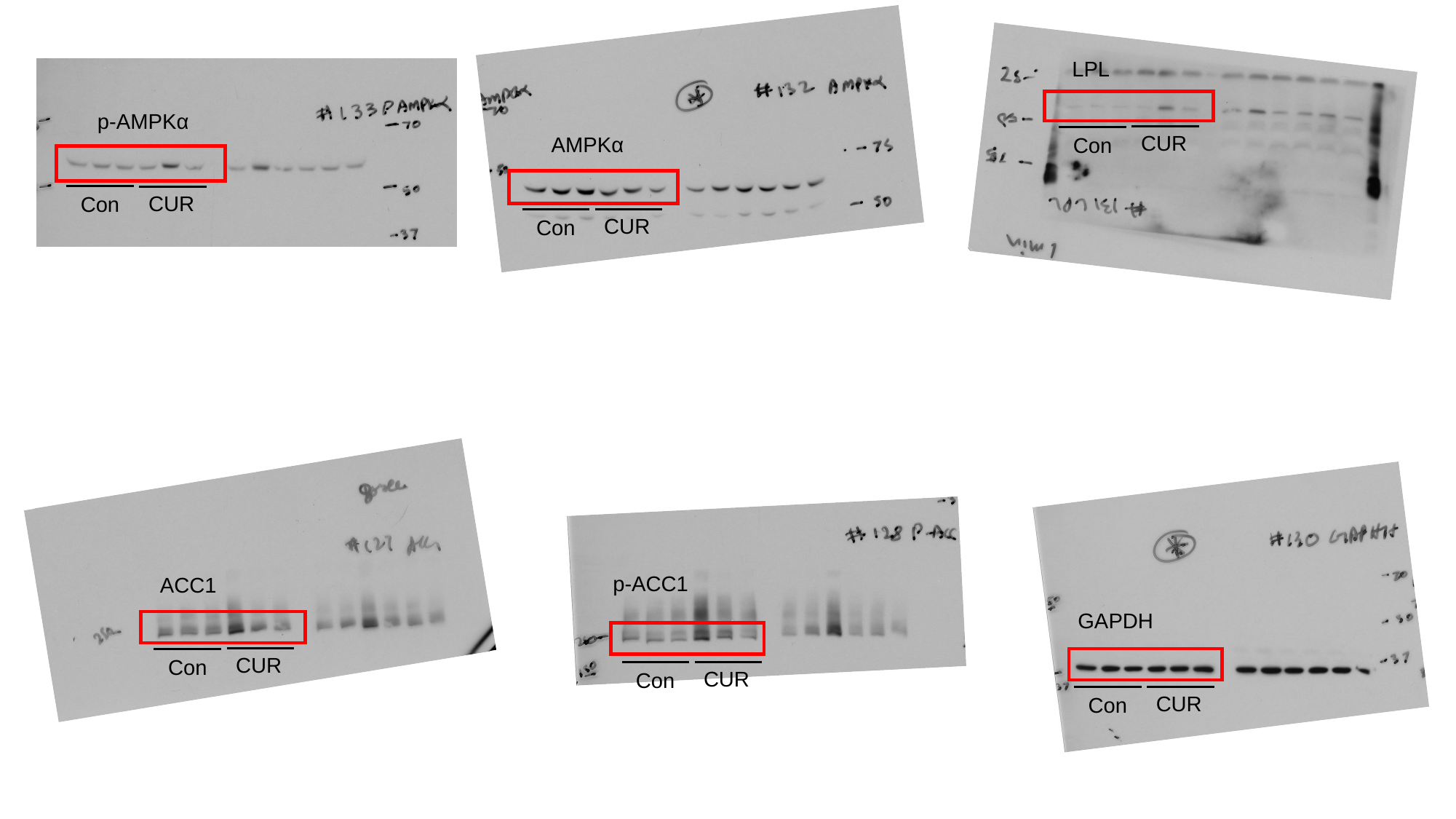

LPL
p-AMPKα
CUR
AMPKα
Con
CUR
Con
CUR
Con
p-ACC1
ACC1
GAPDH
CUR
Con
CUR
Con
CUR
Con
